# Protective effect of soluble eggshell membrane protein hydrolysate on cardiac ischemia/reperfusion injury

**DOI:** 10.3402/fnr.v59.28870

**Published:** 2015-12-21

**Authors:** Tao Yang, Yan Li, Meihu Ma, Qinlu Lin, Shuguo Sun, Bin Zhang, Xi Feng, Junwen Liu

**Affiliations:** 1 National Engineering Laboratory of Rice and By-product Deep Processing, Central South University of Forestry and Technology, Changsha, People's Republic of China; 2 College of Food Science & Engineering, Central South University of Forestry & Technology, Changsha, People's Republic of China; 3 Department of Histology and Embryology, School of Basic Medical Sciences, Central South University, Changsha, People's Republic of China; 4 National R&D Center for Egg Processing, Huazhong Agricultural University, Wuhan, People's Republic of China

**Keywords:** cardioprotection, caspase, ischemia-reperfusion injury, cardiomyocytes, soluble eggshell membrane protein

## Abstract

**Background:**

Soluble eggshell membrane protein (SEP) has been proved to hold the antioxidant activity. The functional role of SEP on cardioprotection was investigated *in vivo* and *in vitro*.

**Methods:**

Rats and cardiomyocytes were pretreated with SP2, a hydrolysate attained from SEP, and then subjected to ischemia/reperfusion (I/R) or hypoxia/reoxygenation (H/R) and hydrogen peroxide, respectively. The measurement of myocardial infarct size, cell apoptosis assay, cell viability assay, and caspase activity assay were performed on rats and cardiomyocytes.

**Results:**

The results showed that the treatment of SP2 induced the resistance to I/R or H/R injury on rats and cardiomyocytes as indicated by decreased infarct size and decreased cellular apoptosis. The cardioprotective roles of SP2 were partly resulted from the downregulated expression and activity of caspase-3 in which the effect was similar to the caspase inhibitor, z-VAD-fmk, and could be rescued by caspase activator, PAC-1.

**Conclusions:**

This investigation has demonstrated that SP2 attenuated the damage of I/R and H/R on rats and cardiomyocytes by the caspase-dependent pathway. This cardioprotective effect of SP2 suggested a novel therapeutic agent of SEP for ischemic-related heart diseases.

Nearly 30% of eggs consumed each year are broken and processed or used in foods such as cakes, mixes, mayonnaise, noodles, and fast foods. The shell is a calcareous structure predominantly constituted of calcium carbonate (CaCO_3_) (95%) and an organic matrix composed of proteins, glycoprotein, and proteoglycans (3.5%) (1, 2). The disposal methods for waste eggshells are 26.6% as fertilizer, 21.1% as animal feed ingredients, 26.3% discarded in municipal dumps, and 15.8% used in other ways (3). Actually, the eggshell and its membranes are an inexpensive and abundant material with a high content of bioactive components for many potential clinical, cosmestic, nutraceutical, and nanotechnology applications (4). However, soluble eggshell membrane protein (SEP) has been studied to a limited extent for disease-preventative characteristics. Our laboratory isolated the SEP from eggshell membrane (ESM) and attained the hydrolysates with the antioxidative activities. Among the hydrolysates, SP2 contains some amino acids (His, Tyr, Met, Cys, Val, Pro, Phe Trp, Leu, Ile, Ala, Asp, and Glu) and was the fraction with the strongest scavenging activity (5).

Heart damage following acute myocardial infarction and ischemia, and other cardiac pathologies, is a major contributor to heart dysfunction and failure (6). Apoptosis of cardiomyocytes has been identified as an important mechanism involved in the initiation and progression of heart failure (7). During this process, oxidative stress due to accumulation of reactive oxygen species (ROS) has been implicated (8, 9). Although the antioxidative activity of SEP has been demonstrated by the chemical analysis (5), the particular effect of SEP *in vivo* and *in vitro* during oxidative stress remains unknown.

In this study, we observed the protective roles of SP2 on the rats and myocardiocytes H9c2 subjected to ischemia/reperfusion (I/R) or hypoxia/reoxygenation (H/R), and investigated the primary molecular mechanism, to provide the certain evidences for SP2 as the potential cardioprotective agent.

## Results

### SP2 attenuated the I/R-induced cardiac injury of rats

**Fig. 1:**
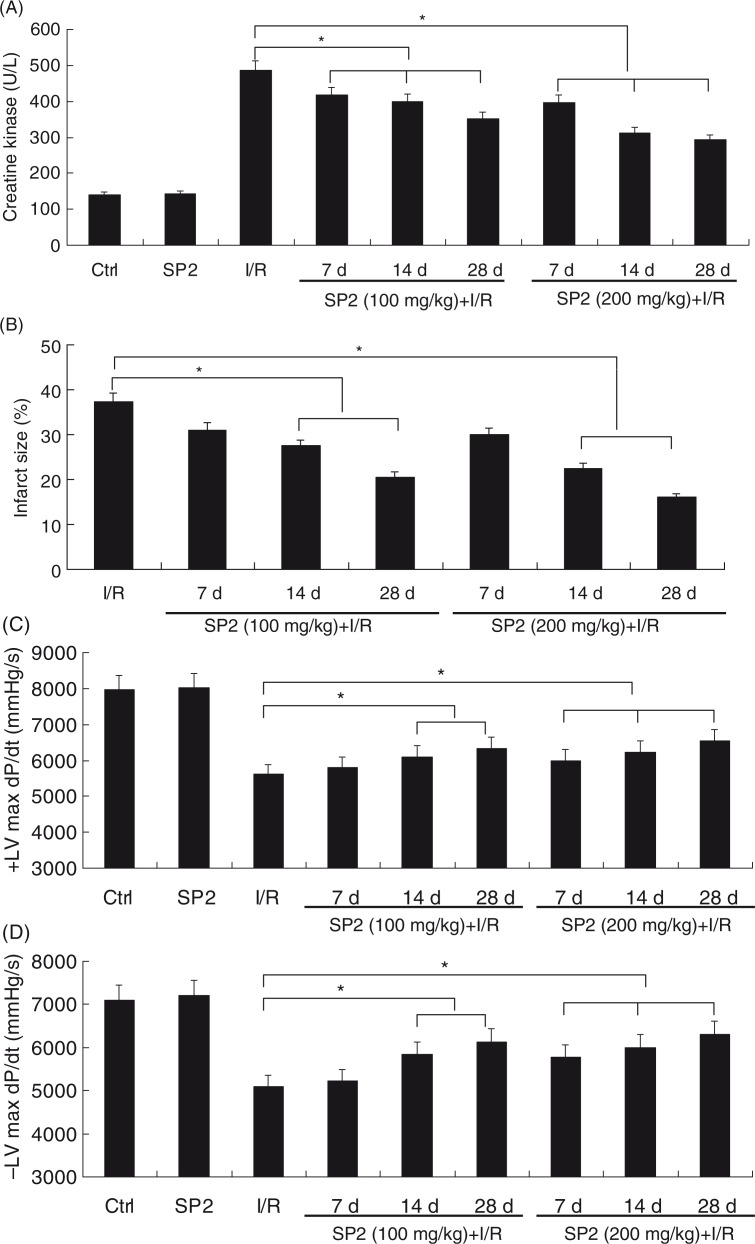
Effect of SP2 on the I/R-induced injury *in vivo*. (A) Change of creatine kinase (CK) activity in the serum of mice. (B) The infarct size (% IAR) in the hearts of mice. (C) The maximal slope of systolic pressure decrement. (D) The maximal slope of diastolic pressure decrement. Rats of the SP2 groups were given SP2 (100 mg/kg and 200 mg/kg body weight; BW) every day for 7 days, 14 days, and 28 days by oral gavage, then subjected to 30-min ischemia followed by 4-h reperfusion. The relative values of all results were determined and expressed as mean±SEM of three experiments. Ctrl: Sham group; SP2: Sham rats pretreated with SP2 (200 mg/kg BW for 28 days, *n*=6). *P<0.05.

To investigate the effect of SP2 on the oxidative stress *in vivo*, we have used a rat myocardial I/R injury model (30-min ischemia followed by 4-h reperfusion) (10). In the SP2 groups, rats were given SP2 (100 mg/kg and 200 mg/kg body weight; BW) every day for 7 days, 14 days, and 28 days by oral gavage, then subjected to I/R injury. As shown in Fig. 1A and B, I/R caused creatine kinase (CK) release and subsequent development of a myocardial infarction *in vivo*, while the treatment of SP2 alleviated the I/R-induced heart damage markedly, especially in the dose of 200 mg/kg. Haemodynamic indices were measured to determine the protective effect of SP2 on the left ventricle function of rats. The I/R-induced decrements of +dP/dt and −dP/dt were significantly attenuated in SP2-treated rats (Fig. 1C and D). Together, these results indicate that the treatment of SP2 gives a protection for I/R-induced myocardial injury.

### SP2 inhibited the H/R-induced and H_2_O_2_-challenged injury of cardiomyocytes

**Fig. 2:**
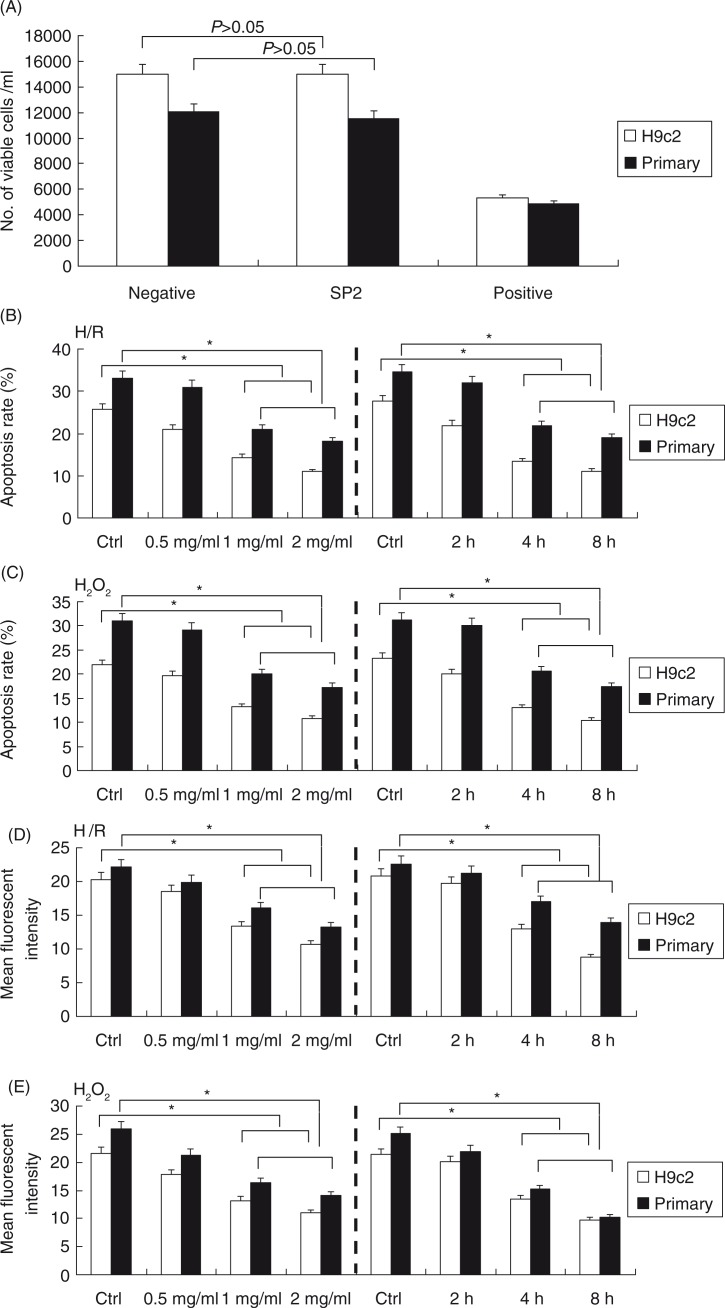
Effect of SP2 on the H/R- or H_2_O_2_-induced injury *in vitro*. (A) Cell viability upon treatment of SP2 (2 mg/ml) was determined by Trypan blue assay. Positive control, genistein (200 µM) in saline with 0.1% methanol; negative control, saline with 0.1% methanol. (B) and (C) Apoptotic rates were determined by flow cytometry. (D) and (E) ROS generation levels were determined by CM-H2DCFDA dye and flow cytometry. The cells of SP2 groups were pretreated with SP2 in different concentrations for 4 h (0.5 mg/ml, 1 mg/ml, 2 mg/ml) or in various periods of time (2 h, 4 h, 8 h) in the concentration of 1 mg/ml before H/R (hypoxia 2 h and reoxygenation 12 h) or H_2_O_2_ challenge (0.5 mmol/L for 12 h). Ctrl: H/R or H_2_O_2_-challenged cells. Before the challenges, the control cells were cultured for 4 h in different concentration groups, or for 8 h in different time groups, respectively. The relative values of all results were determined and expressed as mean±SEM of three experiments. *P<0.05.

Figure 2A showed that there was no toxicity of SP2 on the cultured primary neonatal rat cardiomyocytes and the cell line of cardiomyocytes H9c2, and then the cells were subjected to the hypoxia/reoxygenation (H/R) or H_2_O_2_ injury. The cells of SP2 groups were pretreated with SP2 in different concentrations for 4 h (0.5 mg/ml, 1 mg/ml, 2 mg/ml) or in various periods of time (2 h, 4 h, and 8 h) in the concentration of 1 mg/ml before H/R (hypoxia 2 h and reoxygenation 12 h) or H_2_O_2_ challenge (0.5 mmol/L for 12 h), then the ROS generation levels and apoptotic conditions of cells were determined. Compared with the H/R or H_2_O_2_-challenged cells, the SP2 pretreatment could reduce the cell apoptotic rates of the primary cardiomyocytes and H9c2 cells (Fig. 2B and C), as well as the generation of endogenous ROS (Fig. 2D and E).

### The protective role of SP2 was dependent on the caspase-3 pathway

**Fig. 3:**
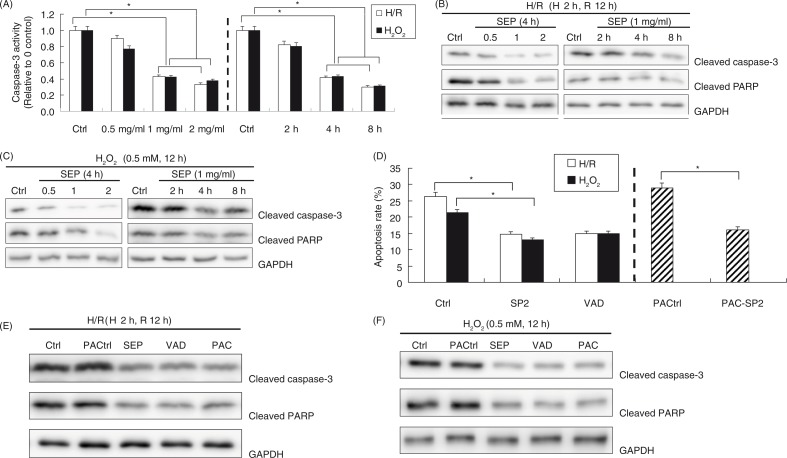
Effect of SP2 on the caspases pathway. (A) The caspase-3 activity was assayed by using caspase fluorescence assay kits. (B) and (C) Expressions of cleaved caspase-3 and PARP were determined by Western blot. The H9c2 cells of SP2 groups were pretreated with SP2 in different concentrations for 4 h (0.5 mg/ml, 1 mg/ml, 2 mg/ml) or in various periods of time (2 h, 4 h, 8 h) in the concentration of 1 mg/ml before H/R (hypoxia 2 h and reoxygenation 12 h) or H_2_O_2_ challenge (0.5 mmol/L for 12 h). Ctrl: H/R or H_2_O_2_-challenged cells. Before the challenges, the control cells were cultured for 4 h in different concentration groups or for 8 h in different time groups, respectively. (D) Apoptotic rates were determined by flow cytometry. (E) & (F) Expression of cleaved caspase-3 and PARP were determined by western blot. Ctrl: H/R or H_2_O_2_-challenged cells; SP2: Cells were pretreated with SP2 (1 mg/ml, 4 h) before H/R or H_2_O_2_ challenge; VAD: Cells were pretreated with z-VAD-fmk (50 µM, 2 h) before the challenges; PACtrl: PAC-1-induced cells (75 µM, 20 h); PAC-SP2: Cells were pretreated with SP2 (1 mg/ml, 4 h) and then induced by PAC-1. The relative values of all results were determined and expressed as mean±SEM of three experiments. *P<0.05.

To investigate the primary molecular mechanism of SP2 on the antiapoptotic effects, we examined the levels of cleaved caspase-3 and cleaved PARP (poly ADP-ribose polymerase), and the caspase activities. The results showed that the pretreatment with SP2 resulted in deactivation of caspase-3 in the H/R and H_2_O_2_-challenged H9c2 cells, as evidenced by the decreased fluorescence in the caspase assay (Fig. 3A), as well as the decreased levels of cleaved caspase-3, and PARP levels subsequently (Fig. 3B and C). In order to determine the involvement of caspases, we applied the broader caspase inhibitor (z-VAD-fmk) and the direct caspase activator (PAC-1). Figure 3D and E showed that the application of z-VAD-fmk reduced the apoptosis in the H/R and H_2_O_2_-challenged H9c2 cells in which the effect was similar to the treatment of SP2. Furthermore, Fig. 3D and F showed the rescuing effect of SP2 in apoptotic cells induced by PAC-1. The above results reveal that the protective role of SP2 on apoptosis is caspase dependent.

## Discussion and conclusions

I/R injury in humans occurs in conditions such as stroke, cardiac arrest, subarachnoid hemorrhage, or head trauma. The maximal tissue damage is observed during reperfusion, which is primarily attributed to oxidative injury resulting from the production of oxygen-free radicals (11). It has been demonstrated that this ROS-induced oxidative stress plays a crucial role in cardiovascular diseases such as ischemic heart disease, atherosclerosis, congestive heart failure, cardiomyopathy, hypertrophy, and arrhythmias (12). In this report, we demonstrated that the SP2 pretreatment could alleviate the oxidative damage to the cardiomyocytes *in vivo* and *in vitro*, suggesting a novel function of SEP for the application in the related fields.

ESM is a natural material consisting of about 64 proteins, which include Type I, V, and X collagen, lysozyme, osteopontin, and sialoprotein (13). In the previous studies, it has been demonstrated that ESM-derived products can inhibit TNF-α production in peripheral blood mononuclear cells and suppress secretion of pro-inflammatory cytokines in serum of LPS-challenged rats (14, 15). Furthermore, SEP holds the strong antioxidative stress activity and can serve as a potential anti-inflammatory supplement for intestinal health via downregulation of pro-inflammatory cytokine expression and boosting T-cells apoptosis to restore immune homeostasis in gut (13, 16). Here, we observed that the application of SP2 reduced the CK release and the infarct size of I/R injury animals. CK is an enzyme expressed by various tissues and cell types. It catalyzes the conversion of creatine and utilizes adenosine triphosphate (ATP) to create phosphocreatine (PCr) and adenosine diphosphate (ADP). Clinically, CK is assayed in blood tests as a marker of damage of CK-rich tissue such as in myocardial infarction (heart attack), rhabdomyolysis (severe muscle breakdown), muscular dystrophy, the autoimmune myositides and acute renal failure. The reduction of CK and the infarct size of I/R rats demonstrated the protection role of SP2 on the myocardial tissue *in vivo*. Furthermore, SP2 could protect the cardiomyocytes from H/R or H_2_O_2_ stimulation in the dose- and time-dependent manners. These results indicate that SP2 has the potential for the research and development (R&D) not only as the anti-inflammatory agent but also the cardioprotective agent.

Cell apoptosis is one of the most important pathophysiological processes during acute myocardial ischemia and the subsequent reperfusion injury. Apoptotic cardiomyocytes were observed particularly in the border zones of infarcted myocardium, whereas very few apoptotic cells were present in the remote non-infarcted myocardium (17). As the proapoptotic factor in the progress of cardiovascular diseases, oxidative stress is regarded as the key step to discover the drug or bioactive substances improving heart function, inhibiting or alleviating ischemic reperfusion injury (18). In this study, we found that SP2 could alleviate the oxidative damage and the following cell apoptosis. Caspases are essential in cells for apoptosis or programmed cell death and have been termed ‘executioner’ proteins for their roles in the cell. There are two types of apoptotic caspases: initiator (apical) caspases (e.g. CASP2, CASP8, CASP9, and CASP10) and effector (executioner) caspases (e.g. CASP3, CASP6, and CASP7). Initiator caspases cleave inactive pro-forms of effector caspases, thereby activating them; while effector caspases in turn cleave other protein substrates within the cell, to trigger the apoptotic process (19). We detected that the pretreatment of SP2 could decrease the caspases activities and downregulated the expressions of cleaved caspases-3 and PARP, which is one of the final target of caspases, suggesting the protective role of SP2 that may be fulfilled through the caspase pathway. Therefore, the broader caspase inhibitor (z-VAD-fmk) and the direct caspase activator (PAC-1) were applied to testify, and the results showed the caspase-dependent effect.

In summary, our study demonstrated the pretreatment of SP2 could alleviate the apoptosis of cardiomyocytes induced by ischemic reperfusion injury or oxidative damage *in vitro* and *in vivo*, and the potential molecular mechanism was involved in the caspase-dependent pathway. This is the first report about the protective effect of SP2 on the cardiomyocytes. Failure of apoptosis is one of the main contributions to tumor development and autoimmune diseases; this, coupled with the unwanted apoptosis that occurs with ischemia or Alzheimer's disease, has stimulated interest in caspases as potential therapeutic targets since their discovery in the mid-1990s. Here the protective function of SP2 on cardiomyocytes supplies a new probability for the development of healthcare food or drug aiming at caspases.

## Experimental

### Preparation, hydrolysis, and purification of SP2

ESM waste, a byproduct of egg manufacturing of China, was obtained by manual peeling from Chinese commercial eggshells and composed of both inner and outer membranes, then it was processed as described previously (5, 20). In brief, raw ESM pieces were suspended in 1.25 M aqueous 3-mercaptopropionic acid in the presence of 10% acetic acid at 90°C for 6 h. The undissolved ESM was mixed with water at a ratio of 1:30 (w/v). After adjustment of the pH to 2.0 with 0.5 M acetic acid, the above mixture was digested with pepsin at an enzyme to substrate ratio of 2% (w/w) and at 37°C for 4 h. The supernatant, collected after the mixture had been centrifuged at 5,000 rpm for 10 min, was mixed with the supernatant of the first reaction. The mixed supernatant was neutralized to pH 5.0 and allowed to stand for 30 min. SP2 was obtained by centrifuging the solution at 5,000 rpm for 10 min and by freeze-drying the sediment. The SP2 then was dissolved in the sterile phosphate-buffered saline (PBS), and the solution of SP2 was filtered (filter membrane aperture: 0.22 µm) for sterilization as the reagent in the further experiments and stored at 4°C.

### Animals

Male Sprague-Dawley rats, and neonatal Wistar rats (1–3 days) used as a source of primary cardiomyocytes, were purchased from the Animal Resource Center of Central South University. All procedures involving animals were approved by the Ethics Committee of Central South University, Medical Institution Animal Care and Research Advisory Committee (Changsha, China).

### Cardiomyocyte culture and hypoxia/reoxygenation treatment

Primary neonatal rat cardiomyocytes were isolated from hearts of 1- to 3-day-old Wistar rats by trypsin digestion as described previously (21). The H/R treatment on cells was performed as described (21). Briefly, hypoxia/reoxygenation injury was achieved by placing the cells in a hypoxia chamber filled with 5% CO_2_ and 95% N_2_ at 37°C in a glucose-free DMEM, and the cells were reoxygenated with 5% CO_2_ and 95% O_2_ for 3–12 h in DMEM containing 10% serum and 5 mM glucose. Cellular oxidative stress was induced by exposure to hydrogen peroxide (H_2_O_2_) (0.5 mM) at different time points. The concentration of H_2_O_2_ was determined spectrophotometrically at 240 nm as described. A 10-mM stock solution was prepared and diluted into the medium.

### Animal models of cardiac I/R injury

Cardiac I/R injury experiments in animals were performed as described previously (21, 22). In brief, the rats were anaesthetized with pentobarbital (70–80 mg/kg ip) every 2 h. Under sterile conditions, the heart was exposed by a left thoracotomy in the fourth intercostal space. I/R was achieved by a 30-min occlusion of the left anterior descending coronary artery (LAD), followed by 4-h reperfusion. The rat received a single intraperitoneal injection of PBS (vehicle) or zinc protoporphyrin-IX (ZnPP, an HO-1 inhibitor) (Sigma-Aldrich, St. Louis, MO) at a dose of 50 µmol/kg, and then underwent I/R. The maximal slope of systolic pressure increment (+dP/dt) and diastolic pressure decrement (−dP/dt) was measured in anaesthetized rat as described. After the haemodynamic measurements, the rats were sacrificed, the blood was collected, and the hearts were excised. CK release was measured by a commercially available kit (Sigma).

### Measurement of myocardial infarct size

After reperfusion, the animals were anaesthetized, the LAD was reoccluded, and 0.6 mL of 10% Evans blue dye (Sigma) was slowly injected into the aorta to define the ischemic zone. The myocardial ischaemic area at risk (IAR) was identified as the region lacking blue staining. Serial, short-axis 1-mm-thick sections were cut and incubated in 2% triphenyltetrazolium chloride (TTC, Sigma) at 37°C for 10 min for demarcation of viable and non-viable myocardium. Positive TTC staining was in red color, and the infarcted area was pale. Images were analyzed by Image-Pro Plus, and infarct size was expressed as a percentage of IAR (% IAR) (21).

### Trypan blue assay for cell viability

Cell viability was detected by the Trypan blue dye exclusion assay as described previously (6). Briefly, the monolayer was allowed to grow for 2–3 days at 37°C on 24 well plates. After the cell treatment, the medium was removed, and the cells were briefly digested with 0.1% trypsin plus 0.53 mM ethylenediaminetetraacetic acid (EDTA) solution. And then, 0.5% Trypan blue dye mixed in growth medium was added to each well. Samples were then aspirated from each well and loaded into chambers in a hemocytometer cell for the further cell counting. This assay reflected the number of viable cells that survived after treatment with SP2 at the highest concentration we have applied in this study.

### Flow cytometry and ROS determination

When all groups of cells were cultured into confluence, cells were collected and washed twice in PBS and then were fixed in 70% ethanol overnight at 4°C. Cell samples were stained with propidium iodide (50 mg/ml; Sigma). The sub-G1 peak was measured by FACScan Flow Cytometry (Becton Dickinson, Franklin Lakes, NJ) and analyzed by Cell Quest software. On the other hand, intracellular ROS were measured fluorimetrically with the CMH2DCFDA probe. The cells were washed with PBS, incubated with 5-µM CM-H2DCFDA at 37°C for 30 min and then washed to remove excess CM-H2DCFDA, and fluorescence was determined at 485-nm excitation and 538-nm emission wavelengths. Relative ROS production is expressed as a change in fluorescence.

### Caspase activity assay

The caspase fluorescent assay kits specific for caspase-3 (Biovision, Mountain View, CA) were used to detect caspase activation by measuring the cleavage of a synthetic fluorescent substrate as described (6). In brief, cells were cultured in 60-mm dishes and treated. Cell lysates were prepared with the lysis buffer provided by the assay kit and centrifuged at 10,000×g for 1 min, and the supernatants were collected. With bovine serum albumin (BSA) as the standard for protein content, equal amounts of protein were reacted with the synthetic fluorescent substrates at 37°C for 1.5 h and the absorbance at 405 nm was read on a microplate reader. Fold increase in caspase-3 activity versus control was determined.

### Western blot analysis

After various treatments, proteins in the whole-cell lysates were resolved on 10% sodium dodecyl sulfate polyacrylamide gel electrophoresis (SDS-PAGE) and then transferred onto polyvinylidene difluoride (PVDF) membranes (Schleicher & Schuell, Dassel, Germany). The membranes were blocked overnight in PBS containing 10% non-fat dry milk and 0.5% Tween-20 and incubated with primary antibodies for 2 h. Horseradish peroxidase-conjugated anti-rabbit or anti-mouse IgG was used as the secondary antibody. The immunoreactive bands were visualized using DAB (Boster Biological Technology, Wuhan, Hubei, China). Glyceraldehyde-3-phosphate dehydrogenase (GAPDH) antibody was used to normalize for equal amounts of proteins and to calculate the relative induction ratio. The following antibodies were used: rabbit monoclonal-cleaved caspase-3 antibody (1:1,000, Cell Signaling Technology, Danvers, MA), rabbit monoclonal-cleaved PARP antibody (1:1,000, Cell Signaling Technology), rabbit polyclonal GAPDH antibody (1:1,000, Abcam, Cambridge, UK), and HRP-conjugated anti-rabbit IgG (1:1,000, Abcam).

### Statistical analysis

Each experiment was performed three times, and the data were expressed as mean±SEM or representative data were shown. Statistical analysis was performed using a two-tailed Student's t-test or Fisher's least significant difference test; otherwise, representative data were shown. *P*<0.05 was considered significant.
